# Comparison of Negative Pressure Wound Therapy Systems and Conventional Non-Pressure Dressings on Surgical Site Infection Rate After Stoma Reversal: Systematic Review and Meta-Analysis of Randomized Controlled Trials

**DOI:** 10.3390/jcm14051654

**Published:** 2025-02-28

**Authors:** Elissavet Anestiadou, Stavros Stamiris, Orestis Ioannidis, Savvas Symeonidis, Stefanos Bitsianis, Konstantinos Bougioukas, Thomas Karagiannis, Efstathios Kotidis, Manousos-Georgios Pramateftakis, Ioannis Mantzoros, Angeliki Cheva, Georgios Geropoulos, Christiana Chatzianestiadou, Magdalini Kaprianou, Freiderikos Tserkezidis, Stamatios Angelopoulos

**Affiliations:** 1 4th Department of Surgery, General Hospital “George Papanikolaou”, Aristotle University of Thessaloniki, 57010 Exochi, Greece; elissavetxatz@gmail.com (E.A.); simeonidissavvas@yahoo.com (S.S.); sbitsiani@gmail.com (S.B.); skotidis@gmail.com (E.K.); mpramateftakis@hotmail.com (M.-G.P.); imanvol@gmail.com (I.M.); cchristia@auth.gr (C.C.); magdarenos@yahoo.gr (M.K.); frtserkezidis@gmail.com (F.T.); saggelopoulos@auth.gr (S.A.); 2 Orthopaedic Department, 424 General Military Hospital, Ring Road West, Nea Efkarpia, 56429 Thessaloniki, Greece; st.stamiris@hotmail.com; 3 Department of Hygiene, Social-Preventive Medicine & Medical Statistics, School of Medicine, Faculty of Health Sciences, Aristotle University of Thessaloniki, 54124 Thessaloniki, Greece; kbugiuk@yahoo.com; 4 Clinical Research and Evidence-Based Medicine Unit, Second Medical Department, Aristotle University of Thessaloniki, 54642 Thessaloniki, Greece; tkaragian@auth.gr; 5 Pathology Department, Faculty of Medicine, Aristotle University of Thessaloniki, 54124 Thessaloniki, Greece; antacheva@auth.gr; 6 Department of General Surgery, Watford General Hospital, West Hertfordshire Teaching Hospitals, Watford WD180DH, UK; geropoulosgeo@gmail.com

**Keywords:** Negative Pressure Wound Therapy (NPWT), Surgical Site Infections (SSI), stoma reversal, meta-analysis, Randomized Controlled Trials (RCTs), colorectal surgery

## Abstract

**Background/Objectives**: Surgical Site Infections (SSIs) rank among the most common complications following stoma takedown and lead to increased morbidity, increased Length of Hospital Stay (LOS), and higher healthcare costs. Negative Pressure Wound Therapy (NPWT) systems have emerged as a promising option for optimizing wound management and minimizing SSI rates. This systematic review and meta-analysis compares postoperative outcomes of NPWT and conventional Non-Pressure Dressings following stoma reversal. **Methods**: A search of the literature published up to 1 September 2024 was conducted across MEDLINE/PubMed, and the Cochrane Central Register of Controlled Trials (CENTRAL), and Scopus, as well as ClinicalTrials.gov. Only Randomized Controlled Trials (RCTs) were included. The primary outcome was SSI rate, while secondary outcomes included time to complete wound healing, LOS, and patient-reported wound cosmesis. Quality assessment was performed using the Cochrane Risk of Bias 2 (RoB 2) tool. The results were synthesized using means and Standard Deviations for continuous variables, counts and percentages for categorical variables, and presented as Odds Ratios (OR) or Mean Differences (MD) with 95% Confidence Intervals, using random or fixed effects models based on heterogeneity (I^2^). **Results**: Six RCTs, including 328 patients, were ultimately eligible for inclusion. No significant difference was revealed in SSI rates between the NPWT and conventional dressing groups (OR = 0.95; 95% CI: 0.27–3.29; *p* = 0.94; I^2^ = 38%). Time to complete wound healing was significantly lower in the NPWT group compared to conventional dressings (MD = −3.78 days; 95% CI: −6.29 to −1.27; *p* = 0.003). Two studies reported a lower rate of wound healing complications other than SSIs in the NPWT group (OR = 0.22; 95% CI: 0.05–1.09; *p* = 0.06). No substantial differences were observed in terms of LOS (MD = −0.02 days; 95% CI: −1.22 to 1.17; *p* = 0.97) and patient-reported wound cosmesis (SMD = 0.31; 95% CI: −0.49 to 1.11; *p* = 0.44). The review’s limitations include potential risk of bias, variability in study designs, and heterogeneity between studies. **Conclusions**: NPWT contributes to improved wound management through reducing wound healing time compared to Non-Pressure Dressings after stoma reversal, although it does not appear to substantially impact SSI rates, LOS, or patient-assessed wound cosmesis. Further large-scale, multicenter RCTs are necessary to validate these results and identify patient populations most likely to benefit from NPWT application.

## 1. Introduction

Colorectal surgical procedures are ranked among the most common operations performed worldwide, due to the significantly increased incidence of both colonic and rectal benign lesions and malignancies [1,2]. In proportion, the construction of intestinal stomas is quite a common surgical procedure, performed to address numerous benign or malignant colorectal conditions, such as such as colorectal malignancy, inflammatory bowel diseases, post-radiation injury, diverticular disease, perineal inflammation and infection, and fecal incontinence, both in emergency and elective settings [3]. Despite the obscure ostomy-related statistical data due to underreporting, approximately 750,000 to 1 million patients in the United States have a stoma, while approximately 150,000 surgical procedures including stoma creation take place each year [4]. The most commonly encountered types of stomas in surgical practice include Hartman’s end colostomy, loop colostomy, and ileostomy [5]. Furthermore, stomas are classified as temporary—which are reversed after resolution or improvement of the initial indication—or permanent [3]. According to data from Nationwide Inpatient Sample, approximately 50,155 patients undergo stoma reversal annually [6].

Surgical Site Infections (SSIs), defined as infections occurring within the first 30 postoperative days, or within the first postoperative year in presence of implants, may affect up to 20% of patients [7]. SSIs present a significant concern and burden in postoperative care, since they have been associated with higher morbidity and mortality rates, prolonged hospital stay and increased healthcare costs [8,9]. Given that the concentration of bacteria in the colon is approximately 10^12^ per gram of contents and that there is high risk of wound contamination with bowel content, it is well understood why patients undergoing colorectal surgical procedures present SSIs in disproportionally higher rates, compared to patients undergoing other surgical procedures [10,11]. In particular, SSIs constitute the most common complication and a major cause of morbidity after stoma reversal (SR-SSI), with reported incidence ranging from 2 to 41% [12]. Numerous risk factors have been associated with SR-SSI, including colostomy, skin closure technique, Crohn’s disease, diverticular disease, increased operative time, history of fascial dehiscence, and thick subcutaneous fat tissue [13,14,15].

Vacuum-Assisted Closure (VAC) therapy, also known as Negative Pressure Wound Therapy (NPWT), constitutes a revolutionary technological advance, aiming at promoting wound healing through providing subatmospheric negative pressure across the wound bed [16]. Several types of NPWT systems are commercially available, including traditional NPWT systems, instillation NPWT systems and Closed Incision Negative Pressure Therapy (ciNPT) devices [17,18]. Numerous reports highlight the efficacy and feasibility of NPWT systems in reducing SSIs after abdominal operations [19,20]. Furthermore, the superiority of NPWT has been also established in grossly contaminated conditions and in the emergency setting, leading to reduced risk of SSI and wound dehiscence [21]. The literature contains encouraging data regarding SSIs rates and the use of NPWT after stoma reversal, mainly derived by cohort and comparative studies [22,23]. However, no previous systematic review and meta-analysis of Randomized Controlled Trials (RCTs) has comprehensively evaluated the comparative effectiveness of NPWT versus conventional Non-Pressure Dressings specifically in stoma reversal.

The primary objective of this systematic review and meta-analysis is to compare the effectiveness of NPWT systems with conventional Non-Pressure Dressings in reducing SSIs following stoma reversal. By synthesizing data from Randomized Controlled Trials, this study aims to provide evidence-based recommendations for optimal wound management strategies in patients undergoing this high-risk surgical procedure.

## 2. Materials and Methods

A systematic review and meta-analysis was performed to investigate the current evidence provided by Randomized Controlled Trials regarding the effect of NPWT systems on surgical wound treatment after ileostomy or colostomy closure in colorectal surgery, compared to Non-Pressure Dressings. This systematic review was conducted without a pre-existing registered protocol and was constructed based upon the guidelines of the Preferred Reporting Items for Systematic Reviews and Meta-Analyses (PRISMA) [24]. Institutional review board approval was not required as the study did not involve any patient data or intervention. The review protocol was not registered. 

### 2.1. Literature Search

A systematic and thorough search of the literature published up to 1st of September 2024 was performed by two independent reviewers (E.A. and S.S.) in MEDLINE/PubMed, the Cochrane Central Register of Controlled Trials (CENTRAL), and Scopus. ClinicalTrials.gov was also investigated for eligible studies. Search for additional eligible studies was performed using the “similar studies” feature in PubMed after identification of relevant studies, as well as manual searching and perusing reference lists of eligible studies. The search strategy for the database of Medline provided through PubMed, including a combination of free-text and Mesh terms, as well as the adjusted search string applied to the other databases, are presented in the Supplementary Material. In addition, the reference lists of the eligible articles were reviewed, using a snowballing approach [25]. No language, geographical, or chronological restrictions were applied in our search.

The studies were initially reviewed independently from the two reviewers, based on their title and abstract, against to the inclusion criteria. Discrepancies were resolved by a third reviewer (O.I.) and reasons for exclusion were recorded for each study. Subsequently, the full text of the studies of interest was obtained and was assessed for inclusion and extractability of content.

### 2.2. Inclusion Criteria

In the present study, we included studies with the following characteristics: (1) Randomized Controlled Trials (RCTs); (2) including adult patients; (3) studying the effect of NPWT systems after stoma (ileostomy or colostomy) closure, either with purse-string or linear skin closure, compared with Non-Pressure Dressings (gauzes, lints, plasters, bandages (natural or synthetic) and cotton wool); and (4) SSI rate among the outcomes.

### 2.3. Exclusion Criteria

The exclusion criteria for the studies in our review were studies concerning pediatric population, experimental or in vitro studies, case reports or case series, retrospective or prospective observational studies, narrative or systematic reviews, comments or editorials, book chapters, studies with non-extractable data or studies whose full-text was not available online or through communication with the authors, abstract publications, and protocols of incomplete clinical trials.

### 2.4. Data Extraction and Quality Assessment

Before data collection, the two reviewers (E.A. and S.S.) built a data extraction sheet and pilot tested it in a representative sample of eligible studies before data collection. Data collected included:

- Study identification data and study characteristics: first author’s name and year of publication, country of origin of first author, trial register ID number, RCT design type, study time frame-duration, funding sources, number of participants randomized;
- Number of study arms included the number of patients per arm, inclusion/exclusion criteria, study quality, type of analysis, deviation from the initial protocol and selective reporting, follow-up period;
- Baseline characteristics of participants (mean age and Standard Deviations (SD), gender, BMI, primary indication for stoma creation, type of ostomy, technique of stoma-skin closure, administration of antibiotics);
- Data for the intervention (type of NPWT, duration of application, NPWT brand, frequency of dressing changes, duration of follow-up);
- Data for the comparator (type of Non-Pressure Dressings, duration of application, frequency of dressing changes, duration of follow-up);
- Outcome data (outcome title, outcome definition and diagnostic criteria, SSI rate, rate of other wound healing complications, days of hospital stay, time to complete wound healing, patient-reported wound cosmesis).

Both reviewers collected and tabulated independently the data of the final papers chosen for this review. After data collection and comparison of retrieved information between the two reviewers, discrepancies were resolved with consultation of a third reviewer (O.I.).

### 2.5. Outcomes

The primary outcome of our study was SSI rate. Our secondary outcomes were Length of Hospital Stay, time to complete wound healing, rate of other wound healing complications, and patient-reported wound cosmesis. All study outcomes were predefined. Regarding other wound healing complications, we defined all wound-related undesired outcomes except from SSIs.

### 2.6. Statistical Analysis

Continuous variables were summarized as means and Standard Deviations (SD), while categorical variables were reported as counts and percentages. For studies that presented medians and interquartile ranges or ranges instead of means and SD, the Wan et al. method was applied to estimate means and SD values [26]. For the primary outcome (SSI rate) and other categorical outcomes (e.g., wound healing complications), results were synthesized as Odds Ratios (OR) with 95% Confidence Intervals (CI). For continuous outcomes, including Length of Hospital Stay and time to complete wound healing, results were reported as Weighted Mean Differences (WMD) with 95% CI when outcomes were measured on the same scale. For outcomes measured on different scales, such as patient-reported wound cosmesis, the Standardized Mean Difference (SMD) was calculated with 95% CI. Statistical heterogeneity was assessed using the I^2^ statistic, with thresholds of <25% indicating low heterogeneity, 25–75% moderate heterogeneity, and >75% high heterogeneity. A *p*-value < 0.05 was considered statistically significant for all analyses. A random effects model analysis was performed when I^2^ was moderate to high (>25%). In cases of low heterogeneity (<25%), a fixed effects model was used. Meta-analyses and forest plots were generated using Review Manager (RevMan) version 5.4.1 software. The studies in the forest plot were ordered by study weight, prioritizing those with the greatest statistical contribution to the overall effect size, ensuring that the most influential evidence is highlighted. For studies including more than 2 intervention arms, it was decided to pool data from the intervention groups into a single group and consider them one intervention category, and weighted averages and pooled variance were calculated to pool two groups’ means and SDs. For studies reporting zero events in one group (Tiang et al. [27]), continuity corrections were applied to facilitate statistical synthesis, while studies reporting zero events in both groups were automatically excluded by RevMan from the analysis. For continuous outcomes, an SD of 0 was encountered in one group in the study of Wierdak et al. [28], indicating no variability in the reported data. To allow for statistical analysis, a small non-zero value of 0.01 was substituted for the zero SD, consistent with standard meta-analytic practices. Sensitivity analyses were performed to assess the impact of this substitution on the pooled estimates and conclusions. Additionally, subgroup analyses and meta-regression were conducted to explore sources of heterogeneity where applicable.

### 2.7. Quality Assessment Methodology

The quality of the included Randomized Controlled Trials (RCTs) was independently assessed by two reviewers (E.A. and S.S.) using the Cochrane Risk of Bias 2 (RoB 2) tool [29], evaluating the risk of bias across five discrete domains as “low risk of bias”, “some concerns”, or “high risk of bias” for each domain. To ensure consistency, the reviewers piloted the RoB 2 tool on a representative sample of included studies before formal evaluation.

## 3. Results

### 3.1. Study Selection Process

**Figure 1:**
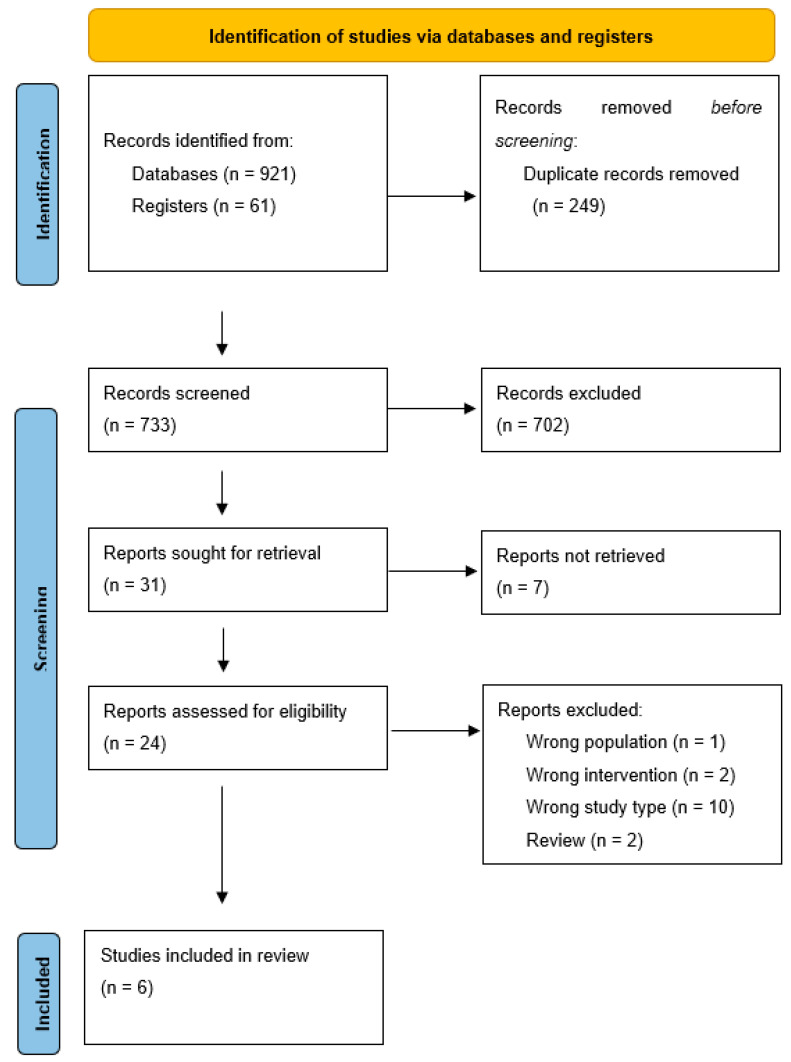
The PRISMA (Preferred Reporting Items for Systematic Reviews and Meta-Analyses) flow chart of the systematic review and meta-analysis.

The initial search of the selected online databases and registers provided a total number of 982 articles. After removal of 249 duplicate records, 733 records were screened based on their title and abstract. After exclusion of 702 records due to low relevance based on title and abstract and inability to retrieve 7 reports, the selection process resulted in 24 articles eligible for full-text analysis, with 6 articles finally accepted for qualitative and quantitative analysis [27,28,30,31,32,33]. A flowchart diagram, according to the PRISMA-P guidelines, is presented in Figure 1 [34]. The present meta-analysis included 328 patients (170 patients in the NPWT group and 158 patients in the conventional Non-Pressure Dressings groups).

### 3.2. Baseline Study Characteristics

A total of six RCTs were included in this meta-analysis, providing an overall number of n = 332 patients for analysis, divided into NPWT and Non-Pressure Dressing groups [27,28,30,31,32,33]. The intervention and comparator groups had a relatively even distribution of participants across studies. The included studies were primarily conducted in Japan (2/6) [30,32], Poland (1/6) [28], Italy (1/6) [31], Korea (1/6) [33], and Australia (1/6) [27], between 2016 and 2024. The pooled mean age of the participants in the present meta-analysis was 58.04 ± 13.49 years for the NPWT and 56.02 ± 14.35 years. Male patients slightly predominated (n = 205), with an approximate male-to-female ratio of 1/0.61. Stoma types were explicitly reported for all patients, with 301 ileostomies and 31 colostomies, created primarily for colorectal cancer (60%) and inflammatory bowel disease (30%). More specifically, four studies included patients who underwent exclusively ileostomy closure [27,28,30,32], while the other two studies analyzed patient who underwent ileostomy or colostomy reversal [31,33]. All studies were designed including two arms, comparing NPWT with Non-Pressure Dressings for wound management after stoma reversal, except for the trial of Kojima et al. [30], which included three arms evaluating Non-Pressure Dressings as well as NPWT application in two different durations, respectively.

Most studies adhered to standardized perioperative antibiotic regimens, predominantly second-generation cephalosporins, with only one study not reporting relevant data [30]. Follow-up durations ranged from 7 to 42 days. NPWT systems used included the PICO Single Use NPWT [30,31,32,33], NANOVA [28], and SNaP^TM^ [27], with pressure settings ranging from −80 to −125 mmHg. In 3out of 6 studies [31,32,33], the diagnosis of SSI was based on the Criteria of the Centers for Disease Control and Prevention (CDC) [35], in one study [28] in a combination of criteria of CDC and European Centre for Disease Prevention and Control (ECDC) [36], in one study [30] based the diagnosis of SSIs on presence of pus in the wound bed, while in one study [27] SSIs are not clearly defined.

Details regarding the baseline characteristics of the studies are portrayed in Table 1 and Table 2, while Table 3 provides a more thorough overview of NPWT systems and Non-Pressure Dressings used in the abovementioned RCTs.

### 3.3. Quality Assessment Outcomes

A quality assessment of included studies was performed in accordance with the revised Risk-of-Bias tool version 2 (RoB 2), provided by Cochrane [29]. A set of five bias domains (D1 to D5) are assessed for discrete aspects of eligible studies, including trial design, conduct or reporting, through answering “signaling questions” for each domain. Answers to these questions provide a rating (“low risk”, “high risk”, or “some concerns”). Two reviewers (A.E. and S.S.) independently assessed the final studies using the methods mentioned above, and possible discrepancies were resolved through a consensus with a third reviewer (O.I.).

**Figure 2:**
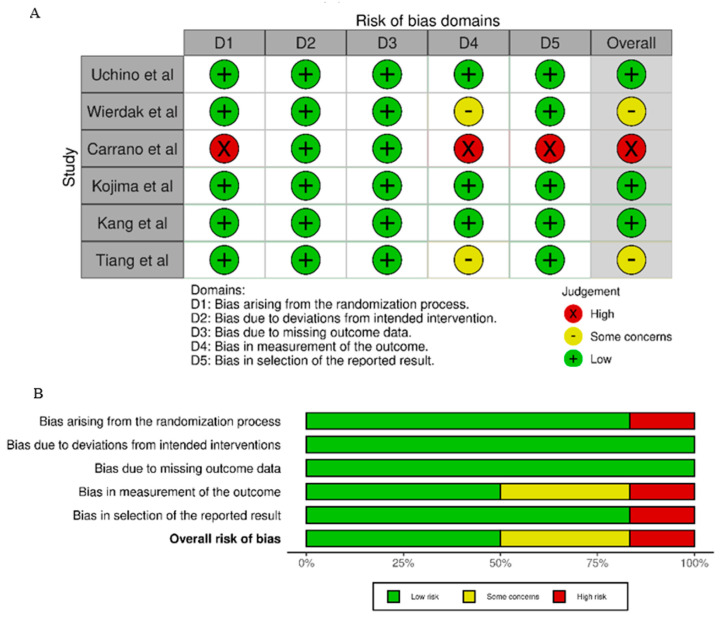
(**A**) Traffic light plot for the domain-specific and overall risk of bias for individual studies [27,28,30,31,32,33] using the ROB2 tool. (**B**) Summary bar chart of the Risk of Bias (RoB) assessment across included studies. The chart illustrates the proportion of studies judged as having low risk (green), some concerns (yellow), or high risk (red) for each domain of the ROB2 tool. The overall risk of bias is also summarized.

Results of quality assessment are presented in Figure 2A,B. Most studies demonstrated a low risk of bias across all domains. The highest proportion of concerns and high risk was observed in “Bias in the selection of the reported result”, suggesting variability in how outcomes were selected and reported. Similarly, “Bias in the measurement of the outcome” presented some concerns in a subset of studies. Carrano et al. [31] was the only study judged to have an overall high risk of bias, with high risks identified in three domains: randomization process, measurement of the outcome, and selection of the reported result. In contrast, the studies of Uchino et al. [32] and Kojima et al. [30] exhibited a consistently low risk of bias across most domains, demonstrating higher methodological rigor. Finally, the studies of Tiang et al. [27] and Kang et al. [33] had some concerns in specific domains but were otherwise assessed as having an overall low risk of bias.

### 3.4. Primary Outcome

**Figure 3:**
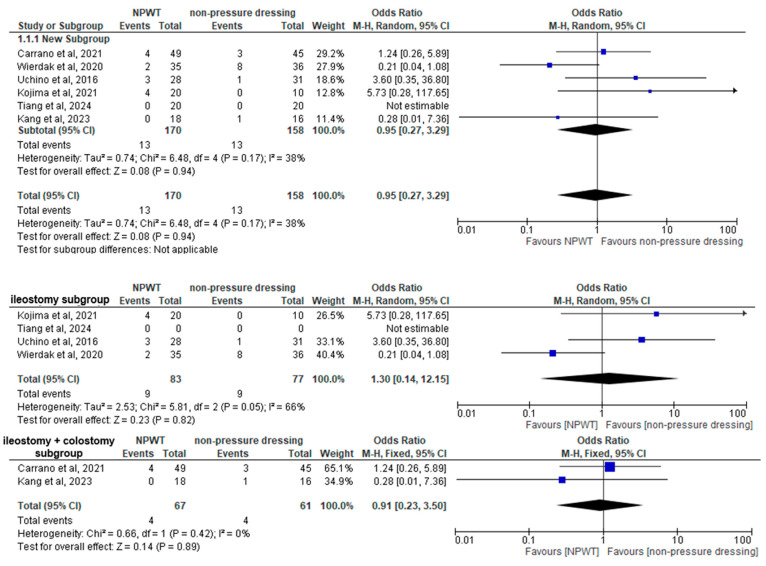
Forest plot showing the difference in SSI rates, both in the whole of the studies, as well as between subgroups [27,28,30,31,32,33].

Six studies, including a total of 328 patients (170 in the NPWT group and 158 in the Non-Pressure Dressing group), were included in the analysis of SSI rate. However, the study by Tiang et al. [27] reported 0 events in both arms, rendering its Odds Ratio non-estimable. For the rest of the studies, the pooled OR for SSI using a random effects model was 0.95 (95% CI: 0.27–3.29, *p* = 0.94), with moderate heterogeneity (I^2^ = 38%) indicating no statistically significant difference between the NPWT and Non-Pressure Dressing groups. Subgroup analysis on the basis of the type of stoma did not alter the results. In detail, the ileostomy subgroup included four studies with a total of 160 patients (83 in the NPWT group, 77 in the Non-Pressure Dressing group) and the ileostomy+colostomy subgroup comprised of two studies with a total of 128 patients (patients (67 in the NPWT group, 61 in the Non-Pressure Dressing group). In both cases, the analyses revealed no significant difference in SSI rates between the two groups ((OR: 1.30; 95% CI: 0.14 to 12.15, *p* = 0.82) and (OR: 0.91; 95% CI:0.23 to 3.50, *p* = 0.89), respectively) (Figure 3).

### 3.5. Secondary Outcomes

**Figure 4:**
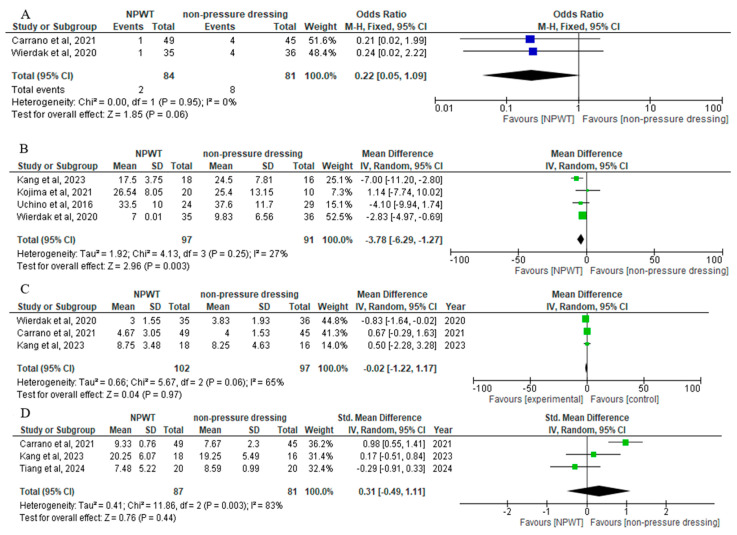
Forest plots showing: (**A**) the difference in rate of wound healing complications other than SSIs [28,31], (**B**) the difference in time to complete wound healing [28,30,32,33], (**C**) the difference in Length of Hospital Stay [28,31,33], (**D**) the difference in postoperative patient-reported wound cosmesis outcomes [27,31,33].

Two studies [28,31], with a total number of 165 patients (84 in the NPWT group, 85 in the Non-Pressure Dressing group), reported the rate of wound healing complications other than SSIs. No significant difference in wound healing complications was reported between the two groups (OR = 0.22; 95% CI: 0.05, 1.09; *p* = 0.06), without heterogeneity (I^2^ = 0%) (Figure 4A).

Four studies [28,30,31,33], with a total number of 188 patients (97 in the NPWT group, 91 in the Non-Pressure Dressing group), reported data on time to complete wound healing. Compared with the Non-Pressure Dressing group, the NPWT group presented a statistically significant reduction of time need to achieve complete wound healing (MD = −3.78; 95% CI: −6.29 to −1.27, *p* = 0.003), with moderate heterogeneity (I^2^ = 27%) (Figure 4B).

Three studies [28,31,33] with 199 patients (102 in the NPWT group, 97 in the Non-Pressure Dressing group), reported LOS outcomes. No significant difference was found between the two groups (MD: −0.02; 95% CI: −1.22 to 1.17, *p* = 0.97), with high heterogeneity (I^2^ = 65%) (Figure 4C).

Three studies [27,31,33] with 168 patients (87 in the NPWT group, 81 in the Non-Pressure Dressing group) reported postoperative patient-reported wound cosmesis outcomes using the Visual Analog Scales (VAS) score or the Patient and Observer Scar Assessment Scale (POSAS) score. No significant difference was found between the two groups (SMD: 0.31; 95% CI: −0.49 to 1.11, *p* = 0.44), with high heterogeneity (I^2^ = 83%) (Figure 4D).

### 3.6. Subgroup Analysis

A subgroup analysis was conducted to explore the impact of stoma type on meta-analytic surgical outcomes. More particularly, on the basis of stoma type, four RCTs that included only patients undergoing ileostomy closure [27,28,30,32] were compared with two RCRs including a mix of ileostomy and colostomy reversals [31,33]. The rationale behind this comparison is arising from the inherent differences between ileostomy and colostomy, particularly in bacterial load and potential for wound contamination, which may influence outcomes such as SSI rates and wound healing time [37]. Studies with mixed populations reported outcomes for both ileostomy and colostomy closures together, which may introduce variability compared to studies focusing exclusively on ileostomy. The findings suggested that the inclusion of colostomy cases did not significantly skew pooled results (ileostomy subgroup OR = 1.30; 95% CI: 0.14, 12.15; *p* = 0.82; I^2^ = 66%, ileostomy+colostomy subgroup OR = 0.91; 95% CI: 0.23, 3.50 q; *p* = 0.89; I^2^ = 0%), but may warrant further investigation to identify potential nuances in mixed populations.

### 3.7. Sensitivity Analysis

A thorough outlier sensitivity analysis was performed to estimate the robustness of the meta-analytic results and to identify potential sources of variability. This involved the sequential exclusion of each individual study at a time, to assess the potential disproportionate impact of any single study on the pooled estimates and heterogeneity metrics. Sensitivity analysis revealed that the exclusion of any single study did not alter the statistical significance of the primary and secondary outcomes.

## 4. Discussion

SSIs represent one of the most commonly encountered postoperative complications after abdominal surgery procedures, with an incidence ranging from 15% to 25%, depending on the level of gross contamination of the wound [38]. In particular, SSIs constitute the most common complication following restoration of enteral continuity during stoma closure, with a reported incidence of 30% after ileostomy closure, while colostomy takedown is more frequently complicated with SSIs due to increased bacterial load [39]. SSIs have been directly associated with increased LOS, readmission rates, healthcare costs, as well as increased mortality rates for specific patient groups, so the establishment and adoption of management practices that aim to reduce SSI is of utmost importance in daily surgical practice [40]. Numerous approaches have been emerged to address this need, including delayed primary wound closure, secondary wound closure, wound bed irrigation with iodine solution, drain placement, and closure of wound with purse-string sutures instead of a linear closure [41,42,43].

With its current form going back in the 1990s, NPWT has provided an impactful effect on healing of acute and chronic wounds [41]. Through application of sub-atmospheric pressure, NPWT systems manage to reduce inflammatory exudate and enhance tissue granulation [44]. Numerous meta-analyses have demonstrative the beneficial effect of NPWT in SSI rate after numerous different abdominal surgical procedures of various degree of contamination [45,46]. However, investigation of SSI rate following stoma closure should be performed with caution due to the inherent high possibility for contamination [47].

To our knowledge, this is the first complete meta-analysis of RCTs examining the effect of NPWT on SSI rate after stoma takedown. All previously conducted systematic reviews with similar object included both observational studies and Randomized Controlled Trials, thus introducing a potential bias due to the inherent limitations of observational studies, such as confounding factors and lack of randomization. More specifically, two previous meta-analyses investigated the outcomes of NPWT application in SSI rate after stoma takedown [48,49]. In 2023, Zhu et al. [48] conducted a comprehensive meta-analysis to evaluate the potential benefit of NPWT application in reducing SSIs following stoma takedown surgeries. The following year, Kisielewski and colleagues [49], in their systematic review and meta-analysis, assessed the effectiveness of Closed Incision Negative Pressure Therapy (ciNPT) compared to primary closure after stoma reversal with pursue-string closure. According to the findings of our meta-analysis, and despite the despite the detailed analysis and rigorous methodology followed, no statistically significant difference in SSI rates arises between NPWT and conventional Non-Pressure Dressings (pooled Odds Ratio (OR) = 0.95; 95% CI: 0.27–3.29, *p* = 0.94). The above result comes in contrast with the trend observed in the literature towards lower SSI prevalence and improved wound healing [50,51]. Furthermore, this result comes in contrast with the results of the meta-analysis conducted by Zhu and colleagues [48], who reported a significant reduction in SSI rates in favor of NPWT (pooled OR = 0.50; 95% CI: 0.29–0.84, *p* = 0.01). However, the latter study, including both RCTs and observational studies, is amendable to selection bias arising from the mixed study design and inclusion of inherent limitations of observational data. By focusing exclusively on RCTs, our review aims to eliminate these confounding factors, providing a more focused evaluation of NPWT’s efficacy. However, the heterogeneity among the included studies was substantial (I^2^ = 31%), indicating notable variability in the study populations, intervention protocols, and outcome definitions. The high heterogeneity observed can be attributed to a series of different factors. First of all, a major source of heterogeneity is the inclusion of studies with mixed populations regarding stoma type (colostomy and ileostomy). Colostomy reversal has been identified as a risk factor for incisional SSI [14]. Despite the fact that both colostomy and ileostomy takedown are always considered contaminated due to opening of gastrointestinal tract, the increased diversity and number of bacteria living in the colon render wound beds after colostomy closure vulnerable to SSI [11,52]. Another source of variability is the different surgical technique selected for stoma closure. Numerous trials and meta-analyses have proved the superiority of purse-string closure over linear skin closure technique during stoma reversal surgeries [53,54]. The latter finding suggests that inclusion of studies with both surgical closure techniques could introduce bias as it potentially reducing the measurable impact of NPWT in patients treated with purse-string closure. Finally, a series of other confounding factors, such as different NPWT devices and application protocols, prophylactic antibiotic administration as well as patient-related factors significantly affect the risk for SSI and may introduce further variability in the meta-analytic findings.

Delayed wound healing is a significant clinical challenge affecting both patient and healthcare system outcomes, as it is closely associated with increased patient morbidity, higher economic burden for healthcare systems, as well as significant hazards for healthcare providers [55]. In terms of sufficient and prompt wound healing, our review found that NPWT significantly reduced wound healing time (MD = −3.78 days; 95% CI: −6.29 to −1.27, *p* = 0.003). This finding comes in accordance with the proved effects of NPWT in the wound healing process, including acceleration of granulation tissue formation, reduction of matrix metalloproteinases, stimulation of angiogenesis, reduction of tissue oedema and exudate, enhancement of blood flow, and limitation of biofilm and local bacterial colonization [56]. The aforementioned conclusion differs significantly from the metanalytic data obtained by Kisielewski and colleagues [49], who found no significant difference in wound healing time between the iNPWT group and the control group (Z = 2.73; *p* = 0.006; χ^2^ = 0.37, df = 1; *p* = 0.54; I^2^ = 0%). This discrepancy may be attributed to difference in NPWT systems applied, since Kisielewski et al. [49] studied the effect of only iNPWT devices on healing dynamics, as well as to the different definitions of wound healing time [51]. Finally, Zhu et al. [48], while acknowledging NPWT’s potential benefit for achieving faster wound healing, did not perform a meta-analysis on this outcome due to inconsistencies in the reported data of included studies.

The evidence drawn from the literature also suggests that NPWT application leads to a reduction of hospitalization days compared with standard dressings, contributing to institutional and financial hospital sustainability [57]. Numerous reports highlight the contribution of NPWT on reduction of hospitalization days [58,59]. On this basis, we would expect the NPWT to have a significantly shorter LOS than the group treated with conventional, Non-Pressure Dressings. However, contrary to the literature trend, our meta-analysis revealed no significant difference in terms of Length of Hospital Stay (LOS) between patients managed with NPWT and those managed with conventional dressings. This finding is consistent with other systematic reviews, including the study of Kisielewski et al. [49], who also concluded that the difference in LOS between treatment groups was not statistically significant (IV = 0.19; 95% CI: −0.66 to 1.04; *p* = 0.76; I^2^ = 0%). Results from the meta-analysis of Zhu et al. [48] also align in the same orientation of no benefit of NPWT systems for Length of Hospital Stay after stoma closure. More particularly, Zhu et al. [48] reported no significant difference in LOS across six trials (n = 688) included in their analysis (MD = −0.16; 95% CI: −0.83, 0.51; *p* = 0.64). However, the latter pooled results were characterized by substantial heterogeneity (I^2^ = 56%). These conclusions suggest that while NPWT may improve localized wound healing, it has not achieved critical influence of broader recovery metrics, such as LOS or overall inpatient recovery time. Given the fact that LOS is a multivariate outcome and is determined by numerous factors, such as patient comorbidities, perioperative care protocols, and institutional discharge policies [60,61]. Furthermore, LOS was not evaluated as a primary outcome in any of the included studies, which may have reduced the sensitivity of the analyses to detect subtle differences between two groups.

Despite the fact that patient satisfaction and favorable cosmetic appearance of the wound scar after stoma closure, represent a crucial issue, reports in the literature evaluating these outcomes are scarce [47]. In terms of patient-reported postoperative wound cosmesis outcomes, data provided from included studies are limited. No significant differences were observed between NPWT and Non-Pressure Dressings (StdMD: 0.31; 95% CI: −0.49 to 1.11, *p* = 0.44). The limited data provided from included studies have been also commented in the meta-analysis of Zhu et al. [48], who admit to have failed in evaluating the esthetic results of intervention and control groups. In addition, drawing safe and definitive conclusions, as represented by the high heterogeneity (I^2^ = 83%), is also complicated by the use of two different patient-based wound assessment tools, the Patient and Observer Scar Assessment Scale (POSAS) [62] or Visual Analog Scales (VAS) [63], which complicates direct comparisons between studies which contributes to the inconsistency of results. Finally, a series of factors, including skin closure technique, surgeon’s expertise and skills, as well as patient-related healing characteristics, which may significantly influence scar cosmesis and mask any potential benefit of NPWT.

## 5. Strengths of the Study

The present study has several strengths, including its comprehensive scope and its focus on RCTs to provide high-quality evidence regarding the role of NPWT systems on wound healing after stoma takedown. More specifically, to the best of our knowledge, the present meta-analysis is the only study to date focusing exclusively on RCTs to evaluate the efficacy of NPWT in stoma reversal procedures. By limiting the analysis to RCTs, it provides the highest level of evidence, eliminating biases commonly associated with observational studies. Additionally, all included RCTs were conducted within the last decade, ensuring that the findings are based on recent advancements in surgical techniques and NPWT technologies, making them highly relevant to contemporary clinical practice. The exclusivity of RCTs enhances the methodological rigor of this meta-analysis, distinguishing it from previous studies that combined observational and randomized data. Furthermore, adherence to the PRISMA guidelines ensures the application of a transparent and standardized approach, through rigorous methodology, including predefined inclusion and exclusion criteria and comprehensive subgroup analyses. The wide range of both clinical-reported outcomes, such as SSI rate, time to complete wound healing, LOS, overall complication rates, and patient-reported outcomes, such wound cosmesis, provides a holistic assessment of NPWT’s role in improving surgical outcomes. Second, the focus on a specific surgical procedure—stoma reversal—ensures the findings are directly relevant to a high-risk patient population. The inclusion of subgroup analyses based on different types of stoma (ileostomy+colostomy or Ileostomy) adds further depth to the findings, while the use of the Cochrane risk of bias tool assures limitation of bias.

## 6. Limitations and Future Directions

The evidence in this study is limited by several factors that must be acknowledged. The most substantial limitation to this paper is the heterogeneity found among the included studies in certain outcomes, such as SSI rates and LOS, indicating a significant variability in the sample populations, intervention and control protocols, and surgical techniques and, thus, eliminating the statistical power of the analysis and limiting the ability to detect a definitive effect of NPWT on SSI rates. Therefore, the high level of heterogeneity observed limits the generalizability of the findings. Additionally, the robustness of our meta-analytic results should be interpreted with caution due to the relatively small number of included RCTs and their moderate-to-high risk of bias in certain domains. Furthermore, variability in outcome definitions and scoring methods, particularly for patient-reported wound assessment, render direct comparisons across studies more complicate. Additionally, the inclusion of only RCTs with exclusion of observational studies, while ensuring methodological rigor, may limit the scope of the analysis. Important results with greater generalizability may be drawn by future trials that will address these limitations by standardizing study protocols and focusing on specific subgroups. The above observation is clearly stated also by Zhu et al., who emphasized the need for more high-quality RCTs to confirm the findings of their meta-analysis and to eliminate the limitations associated with mixed study designs.

## 7. Conclusions

This meta-analysis focuses exclusively on the effect of NPWT on SSI rates after stoma reversal using results from Randomized Controlled Trials. While NPWT did not significantly reduce SSI rates or LOS compared to conventional dressings, it demonstrated a beneficial impact on time needed for complete wound healing, reflecting its potential to improve circumstances for local recovery. The variability in findings highlights the influence of factors such as wound closure techniques and patient characteristics on NPWT outcomes. Future research should be orientated towards large-scale, multicenter RCTs with standardized protocols to identify patient populations undergoing stoma closure most likely to benefit from NPWT and to clarify its role in optimizing postoperative wound management strategies.

## Figures and Tables

**Table 1:** Baseline characteristics of included studies.

Study	Journal	Country	Study Period	Number of Arms	Number of Participants			Lost in Follow-Up	Follow-Up (Days)	SSI Diagnosis Criteria	Outcomes
					Total	NPWT	NPD				
Uchino et al., 2016 [32]	Digestive Surgery	Japan	November 2014–September 2015	2	59	28	31	2 (1 per arm)	28	CDC	Duration of complete wound healing incisional SSI prevalence
Wierdak et al., 2020 [28]	Tech Coloproct	Poland	January 2016–December 2018	2	71	35	36	4 (1 from Non-Pressure Dressing group and 3 from in NPWT group)	30	CDC+ECDC	Healing complications, incidence of SSI, LOS, time to complete wound healing
Kojima et al., 2021 [30]	BMC Surgery	Japan	March 2018–March 2019	3 A: NPD B:NPWT for 7 d, C:NPWT for 14 d	30	20	10	Not specified	1, 3, 7, 10, 14	Presence of pus in the wound	Rate of wound reduction, SSI incidence, wound size healing time, and complication rate (excluding SSI)
Carrano et al., 2021 [31]	BJS Open	Italy	June 2019–January 2021	2	98	50 (finally analyzed 49)	48 (finally analyzed 45)	4 (3 from the Non-Pressure Dressing group and 1 from the NPWT group)	30	CDC	Rate of SSIs and other wound complication SSIs, postoperative wound pain, rate of wound healing after 30 days, wound aesthetic satisfaction
Kang et al., 2023 [33]	Ann Surg Treat Res	Korea	June 2019–May 2021	2	34	18 (finally analyzed 16)	18	2 from the Non-Pressure Dressing group	Twice a week until complete wound healing	CDC	Complete wound-healing period, SSI rate, LOS, total cost, and the Patient and Observer Scar Assessment
Tiang et al., 2024 [27]	ANZ Journal of Surgery	Australia	June 2018–December 2021	2	40	20 (finally analyzed 19)	20 (finally analyzed 19)	1 per arm	7, 14, 42	Not specified	Complete wound healing at day 42, patient-reported wound cosmesis, SSI rate

CDC: criteria of the Center for Disease Control; ECDC: European Centre for Disease Prevention and Control; LOS: Length of Hospital Stay; SSI: Surgical Site Infection; NPWT: Negative Pressure Wound Therapy, NPD: Non-Pressure Dressing.

**Table 2:** Participant demographics and clinical characteristics.

Study	Age (Years)		BMI (kg/m^2^)		Sex (F/M)		Type of Stoma	Stoma Indication	Stoma Closure Technique	Perioperative Antibiotics
	NPWT Group	NPD Group	NPWT Group	NPD Group	NPWT Group	NPD Group				
Uchino et al., 2016 [32]	48.1 ± 14.9	40.4 ± 15.9	19.8 ± 4.3	19.7 ± 3.8	11/17	8/23	ileostomy	Ulcerative colitis, with a 2-stage procedure of after restorative proctocolectomy with ileal pouch anal anastomosis	PSC	Second-generation cephalosporin (20 mg/kg)
Wierdak et al., 2020 [28]	61.6 ± 11.3	62.4 ± 11.3	26.2 ± 4.5	26.2 ± 4.3	11/24	16/20	ileostomy	Colorectal cancer	LC	Second-generation cephalosporin (20 mg/kg)
Kojima et al. 2021 [30]	66 ± 10	64.5 ± 9.81	20.96 ± 2.39	22.72 ± 3.95	8/12	4/6	ileostomy	Not specified	PSC	Not specified
Carrano et al., 2021 [31]	56.32 ± 12.92	55.08 ± 16.25	23.81 ± 3.38	23.45 ± 3.66	15/35	17/31	NPWT group: 41 ileostomy, 9 colostomy NPD group: 38 ileostomy, 10 colostomy	NPWT group: Malignant disease 28/Benign disease 22 Non-Pressure Dressing group: Malignant disease 26/Benign disease 22	PSC	Cefazolin (20 mg/kg)
Kang et al., 2023 [33]	65.75 ± 15.85	61.5 ± 12.97	23.45 ± 21.27	25.35 ± 14.56	9/9	8/8	NPWT group: 10 ileostomy, 8 colostomy NPD group: 12 ileostomy, 4 colostomy	Not specified	PSC	2nd generation cephalosporin
Tiang et al., 2024 [27]	55.67 ± 17.1	62 ± 15.1	27.62 ± 6.21	26.12 ± 4.50	equal number of males and females in each group		ileostomy	NPWT group: Malignant disease 11, Benign disease 9 Non-Pressure Dressing group: Malignant disease 14, Benign disease 6	PSC	Cefazolin and metronidazole

NPWT: Negative Pressure Wound Therapy; PSC: purse-string closure, LC: linear closure; Continuous variables are indicated as mean ± SD.

**Table 3:** Technical details of NPWT and Non-Pressure Dressings.

Study	NPWT Group (Intervention)			Non-Pressure Dressing Group (Comparator)	
	Type	Pressure Settings	Details and Duration	Type of Dressing	Application Protocol
Uchino et al., 2016 [32]	PICO Single Use Negative Pressure Wound Therapy System (Smith and Nephew Healthcare, Hull, UK)	−80 ± 20 mm Hg	24 h after surgery, continued for 2 weeks, with changes every 3–4 days	Simple adhesive plaster	Not specified
Wierdak et al., 2020 [28]	NANOVA negative-pressure dressing	Not specified	Removal of the NANOVA dressing at 72 h, placement of Steri-Strips and a standard sterile dressing, which was then changed every 24 h until the removal of sutures	Sterile wound dressing	1st dressing change at 48 h, and thereafter every 24 h until the removal of sutures
Kojima et al., 2021 [30]	PICO Single Use Negative Pressure Wound Therapy System (Smith and Nephew Healthcare, Hull, UK)	−80 mm Hg	Insertion of foam and PREVENA system on POD 1 day. On POD 3, removal of dressing and foam, washing of the wound and application of NPWT without inserting a foam piece. Continuation up to POD 7/POD 14 and thereafter daily gauze changes until epithelialization	Simple gauze	Removal on POD 1 daily washing of the wound and gauze changes until epithelialization
Carrano et al., 2021 [31]	PICO Single Use Negative Pressure Wound Therapy System (Smith and Nephew Healthcare, Hull, UK)	−80 ± 20 mm Hg	Direct application on the wound, maintenance for 7 days	Iodoform gauze	Packing with iodoform gauze for 48 h, thereafter no packing
Kang et al., 2023 [33]	PICO Single Use Negative Pressure Wound Therapy System (Smith and Nephew Healthcare, Hull, UK)	Not specified	Changes twice a week	Simple transparent waterproof dressing (Allevin, Smith & Nephew Healthcare)	Daily changes
Tiang et al., 2024 [27]	SNaPTM (Smart Negative Pressure) wound care system (Spiracur, Inc., Sunnyvale, CA, USA),	−125 mmHg	A gauze interface wick dressing, cut into a 2.5 cm width strip, with a length 4 times the depth of the wound, was placed in the wound bed, followed by a hydrocolloid dressing connected to 60 mL cartridge. First change of the NPWT system at 3rd post-operative day, complete removal at 7th day	Simple, non-woven, absorbent cotton dressing	Daily changes

h: hours, POD: postoperative day; NPWT: Negative Pressure Wound Therapy.

## Data Availability

Data are available to any qualified researchers upon request to elissavetxatz@gmail.com.

## Supplementary Materials

The following supporting information can be downloaded at: https://www.mdpi.com/article/10.3390/jcm14051654/s1, Document S1: Search strategy

## Abbreviations

CDC	Centers for Disease Control
CENTRAL	Cochrane Central Register of Controlled Trials
CI	Confidence Interval
ECDC	European Centre for Disease Prevention and Control
LOS	Length of Hospital Stay
MD	Mean Difference
NPWT	Negative Pressure Wound Therapy
NPD	Non-Pressure Dressing
OR	Odds Ratio
PICO	Patient, Intervention, Comparison, and Outcome
POSAS	Patient and Observer Scar Assessment Scale
PRISMA	Preferred Reporting Items for Systematic Reviews and Meta-Analyses
RCT	Randomized Controlled Trial
SD	Standard Deviation
SMD	Standardized Mean Difference
SR-SSI	Stoma Reversal Surgical Site Infection
SSI	Surgical Site Infection
VAS	Visual Analog Scale
