# Supplementary material for: Comparison of Negative Pressure Wound Therapy Systems and Conventional Non-Pressure Dressings on Surgical Site Infection Rate After Stoma Reversal: Systematic Review and Meta-Analysis of Randomized Controlled Trials
Source: J Clin Med. 2025 Feb 28;14(5):1654. doi: 10.3390/jcm14051654 (PMC11900534; doi:10.3390/jcm14051654)
Supplement: Supplementary file 1 [file jcm-14-01654-s001.zip › jcm-3471671-supplementary.pdf]

Search Strategy

- Detailed search strategy for the database of Medline provided through PubMed:

Search terms for the condition of interest:

#1 "surgical stomas"[MeSH Terms]

#2 "Ileostomy"[MeSH Terms]

#3 "Colostomy"[MeSH Terms]

#4 "Ostomy"[MeSH Terms]

#5 "Ileostomy"[All Fields]

#6 "Colostomy"[All Fields]

#7 "Ostomy"[All Fields]

#8 "surgical stomas"[All Fields]

#9 “stoma”[All Fields])

#10 (#1 OR #2 OR #3 OR #4 OR #5) AND (#6 OR #7 OR #8 OR #9)

Search terms for the intervention of interest:

#11 "Negative-Pressure Wound Therapy"[MeSH Terms]

#12 “negative pressure wound therap*”[All Fields]

#13 “negative pressure wound dressing*”[All Fields]

#14 “NPWT”[All Fields]

#15 “vacuum-assisted closure”[All Fields]

#16 “vacuum-assisted wound closure”[All Fields]

#17 “VAC”[All Fields]

#18 (#11 OR #12 OR #13 OR #14 OR #15 OR #16 OR #17)

Search terms for condition AND intervention:

#19 (#10 AND #18)

The final search string is developed as following: ("surgical stomas"[MeSH Terms] OR "Ileostomy"[MeSH Terms] OR "Colostomy"[MeSH Terms] OR "Ostomy"[MeSH Terms] OR "Ileostomy"[All Fields] OR "Colostomy"[All Fields] OR "Ostomy"[All Fields] OR "surgical stomas"[All Fields] OR "stoma"[All Fields]) AND ((“negative pressure wound therap*”[All Fields] OR “negative pressure wound dressing*”[All Fields] OR “NPWT”[All Fields] OR “vacuum-assisted closure”[All Fields] OR “vacuum-assisted wound closure”[All Fields] OR “VAC”[All Fields]).

- Detailed search strategy for the database of Scopus:

("surgical stomas" OR ileostomy OR colostomy OR ostomy OR "surgical stomas" OR ileostomy OR colostomy OR ostomy OR stoma) AND (reversal OR closure OR takedown) AND ("negative pressure wound therap*" OR "negative pressure wound dressing*" OR npwt OR "vacuum-assisted closure" OR "vacuum-assisted wound closure" OR vac)

- Detailed search strategy for the database of Cochrane Central Register of Controlled Trials (CENTRAL):

("surgical stomas" OR "ileostomy" OR "colostomy" OR "ostomy" OR "stoma") AND ("negative pressure wound therapy" OR "negative pressure wound dressing" OR "NPWT" OR "vacuum-assisted closure" OR "vacuum-assisted wound closure" OR "VAC")
